# Qualitative analysis of pain impact in adult patients with X-linked hypophosphatemia

**DOI:** 10.1093/jbmrpl/ziaf012

**Published:** 2025-01-23

**Authors:** Nicole Nishime, Christopher Theriault, Richard Feinn, Carolyn M Macica

**Affiliations:** Frank H. Netter MD School of Medicine, Department of Medical Sciences, Quinnipiac University, North Haven, CT 06473, United States; Connecticut Children’s Research Institute, Department of Research Operations and Development, Hartford, CT 06106, United States; Frank H. Netter MD School of Medicine, Department of Medical Sciences, Quinnipiac University, North Haven, CT 06473, United States; Frank H. Netter MD School of Medicine, Department of Medical Sciences, Quinnipiac University, North Haven, CT 06473, United States; Connecticut Children’s Research Institute, Department of Research Operations and Development, Hartford, CT 06106, United States

**Keywords:** X-linked hypophosphatemia, pain, musculoskeletal pain, quality-of-life, qualitative research, chronic limitation of activity, healthcare disparities, social and emotional burden

## Abstract

We previously reported pain as a predominant finding in adult patients with X-linked hypophosphatemia (XLH). This study explored health-related quality of life using the 36-Item Short Form Survey Instrument (SF-36v1) and pain experiences of adults with XLH through qualitative analysis of interviews with 15 patients (11 females, 4 males). Age-adjusted differences using SF-36 were lower than the general population in all domains of health-related quality of life, with significant differences notably related to physical function and pain. Ten themes emerged from semi-structured interviews: (1) chronic and variable pain: patients reported persistent pain of varying intensity and location; (2) impact on mobility and daily life: pain severely restricted daily activities, affecting lifestyle, employment, and independence; (3) pain management: patients used medications, physical therapy, lifestyle changes, alternative therapies, and assistive devices; (4) healthcare and accessibility challenges: access to appropriate care and treatments was limited by insurance issues and healthcare providers’ lack of XLH knowledge; (5) emotional and psychological impact: chronic pain and limitations led to social isolation, depression, and emotional burden; (6) desire for improved information and support: patients sought better information, treatment, and community support; (7) challenges with physical inactivity: staying active was essential but difficult, as inactivity worsened pain; (8) navigating healthcare services: accessing therapy and insurance was often challenging; (9) long-term outlook and concerns: patients worried about disease progression and future health; and (10) advocacy and awareness: increased public and medical awareness was needed to improve care. Although pain is not a universal experience within this population, it is a significant issue for many individuals with XLH. These data illustrate the profound impact of XLH on multiple life aspects underscoring the need for effective pain management strategies, better healthcare accessibility, supportive tools, and a more knowledgeable medical community to improve quality of life. Insights from this study will guide the development of a digital pain self-management intervention tailored to XLH patients to address these needs.

## Introduction

X-linked hypophosphatemia (XLH) is a rare genetic disorder caused by an inactivating mutation in the phosphate-regulating endopeptidase X-linked (PHEX) gene, resulting in elevated levels of the hormone fibroblast growth factor-23 (FGF23). FGF23 suppresses 1,25(OH)2D3 production and dietary phosphate absorption and directly inhibits renal phosphate reabsorption. Consequently, increased levels of FGF23 result in phosphate wasting, hypophosphatemia, and hyperphosphaturia.^1^ Phosphate and calcium are required for proper acquisition, biomineralization, and maintenance of bone, teeth, and auditory ossicles. Because of this, XLH manifests systemically, and some common symptoms in adults may include osteomalacia, abnormal dentin in teeth and dental abscesses, arthralgia, myalgia, arthritis, short stature, and hearing loss.^2–7^

Paradoxically, a hallmark manifestation of XLH is a mineralizing enthesopathy, or the formation of bone spurs (enthesophytes) at fibrocartilaginous tendon and ligament insertion sites.^5^^,^^8^^,^^9^ These enthesophytes can occur in any fibrocartilaginous insertion in the body, including the spine, resulting in decreased range of motion (ROM) and pain. In addition to persistent osteomalacia, enthesophytes advance into adulthood, with both prevalence and number of sites affected increasing with age.^9^ Degenerative osteoarthritis/osteoarthrosis (OA) affects millions of the aging adult population in the United States^10^ and is recognized as a comorbidity of adult XLH.^3–5^^,^^11–14^ Prior studies have shown that adult XLH is characterized by progressive musculoskeletal comorbidities including early-onset, pervasive degenerative OA with marginal osteophytes and subchondral sclerosis.^1^^,^^2^^,^^8^ OA significantly impacts activities of daily living (ADLs) and physical function, and results in a pain-limited ROM in this population.^5^^,^^12^^,^^15^^,^^16^ Western Ontario and McMaster Universities Osteoarthritis Index (WOMAC) osteoarthritis scores confirm the painful and disabling impact of OA in adults with XLH.^17^ Other contributing factors to pain and quality-of-life issues include dental caries and abscesses, common in XLH due to mineral defects in dentin and enamel.^18^^,^^19^ In addition, bone pain has been identified as a significant source of pain and one that correlates with the severity of osteomalacia. Bone pain has been described as both localized and global, with 97% of adults reporting bone pain in a study of 232 adult XLH patients. This finding was further bolstered by data from the Brief Pain Inventory, showing that pain interfered with daily functions.^17^

The FDA approved burosumab, a neutralizing FGF23 mAb, as the first biological therapy for XLH in 2020. In adults, this medication was shown to improve serum chemistries including phosphate, 1,25(OH)2D3, and PTH levels, as well as fracture healing, and improvement in stiffness, physical function, and pain scores. Importantly, it was noted the comorbid and painful features of the adult disease that are not related to osteomalacia or fractures continue to persist.^5^^,^^20–23^ The progressive nature of XLH means that many adults with XLH are burdened with the need to accommodate pain and limited mobility on a daily basis, with few options to manage symptoms. For many rare diseases, patients are often tasked with becoming more knowledgeable about their disease than the nonspecialist physicians who treat them, impeding care for pain-related symptoms as early as childhood.^21^ This lack of knowledge can lead to delays in specialty care, which can worsen disease progression and contribute to pain-related diminished quality of life.^24^ Despite pain being a symptom of many rare musculoskeletal and neuromuscular diseases due to the comorbid features of these disorders, the lack of controlled trials evaluating pain in rare diseases often results in inappropriate or ineffective pain management.^25^ Access to pain-management providers and the difficulty in treating and managing chronic pain are contributing factors that can impact quality of life and functional status in rare disorders.^26^ Thus, it is paramount to consider alternative interventions for patients with XLH that can help individuals manage pain.

Although the physical manifestations and pain surveys have been documented in adults with XLH, the creation of alternative interventions is hindered by the lack of data related to the experience of pain in this population, interest in the use of nonpharmacological approaches to managing pain, and what attributes of a mobile application they may find useful. The goal of this mixed-methods qualitative study was to address the knowledge gap related to pain in adults with XLH to better inform the creation of a pain management tool.

## Materials and methods

### Study design

This study was approved by the Human Experimentation Committee/Institutional Review Board (HEC/IRB) at Quinnipiac University and informed consent obtained from the participants. This study employed a mixed-methods qualitative research study design using semi-structured one-on-one interviews to explore the pain experience of individuals living with XLH. A retrospective intake survey and the SF-36v1instrument were also administered and available in English and Spanish.

### Inclusion and exclusion criteria

Adult subjects were recruited and consented with support from the XLH Network, Inc. and who met the following criteria: (a) 30 yr of age or older with confirmation of an XLH diagnosis using the following criteria: confirmed PHEX gene mutation or variant of unknown significance in the patient or a family member with appropriate X-linked dominant inheritance,^27^ (b) able to speak and understand English or Spanish, (c) currently and consistently taking over-the-counter or prescription medication for pain, and (d) willing and able to engage with a smartphone application to complete online measures. Individuals were excluded if they (a) did not meet the criteria for an XLH diagnosis,^27^ and (b) were not currently taking pain medication for XLH-related pain.

### Intake survey and SF-36

All participants were asked to complete an intake survey. The survey was created by the research team to provide a comprehensive view of the physical, emotional, and functional challenges faced by patients with XLH, as well as the strategies they use for pain management and the impact on daily life. To gain more insight into individual experiences and quality of life, participants were also asked to complete the 36-Item Short Form Survey Instrument version 1 (SF-36v1). The SF-36 looks at health-related quality of life across several domains: physical functioning, role limitations due to physical and emotional health, energy/fatigue, emotional well-being, social functioning, pain, and general health. Data were reported as age-adjusted difference relative to the general national age-matched population,^28^ such that a value of zero indicates no difference between populations.

Age-adjusted SF-36 scores of XLH patients were compared with national norms using one-sample t-tests with an α level for statistical significance set at 0.05. Data are expressed as mean ± SD and clinically meaningful differences determined by a Cohen’s *d* (≥0.8). Statistical analysis was conducted using SPSS (IBM Corp. Released 2023. IBM SPSS Statistics for Windows, Version 29.0.2.0 Armonk, NY).

### Semi-structured interviews

Interview questions included open-ended questions on pain symptoms and their impact on daily life, symptom management strategies, challenges related to their XLH diagnosis, and what features they would find useful in a self-management pain application. Interviews were conducted by members of the research team using a semi-structured format. When necessary, the interviewers asked follow-up questions to gain more insight into participants’ experiences or gain additional clarity on answers.

Individuals who responded to the study recruitment announcement were contacted via email to complete a demographic and intake survey, confirming eligibility and meeting inclusion criteria, as well as to discuss study participation. Interviews for 15 eligible participants were scheduled, and a secure weblink was sent to each. Before their one-on-one virtual interview on Zoom, participants were contacted and provided verbal and written consent. Interview questions were also shared in advance for review. All participants were informed that participation in the study was voluntary and that they could stop at any time, without consequence. Participants also consented to recording and transcription of their interviews. Transcriptions were compared with audio recordings for accuracy before undergoing qualitative analysis by the research team.

### Qualitative analysis

Thematic analysis was employed to identify and interpret patterns and themes. This approach allowed for a comprehensive understanding of the varied experiences of individuals living with XLH and the identification of common themes across different contexts and individuals. Data transcripts of interviews were imported individually into NVivo software, version 1.7.2 (Lumivero, Denver, CO), and an initial coding phase was conducted, where relevant information was highlighted. Initial codes were assigned based on recurring concepts present in the interviews. All interviews were analyzed, although data saturation between interviews was achieved within the first 5 interviews and determined based on evidence that the patient narrative became similar and consistent with common recurring themes. These codes were then grouped into preliminary themes. The themes were subsequently reviewed and refined to align with key insights and the study’s objectives. Following refinement, themes were organized into broader categories to facilitate interpretation and establish a hierarchical structure for clarity in subsequent analyses. Data checks on a subset of transcribed interviews were also performed manually and using HyperRESEARCH (Version 4.5.4, Computer Software, Researchware, Inc., 2022, <http://www.researchware.com/>) to confirm the fidelity of the qualitative analysis. Data were found to be consistent between both platforms.

## Results

### Demographics and intake data

Demographic data for the 15 enrolled participants are summarized in Table 1. Participants were predominantly female and White, with a mean age of 49.1 yr. Most were diagnosed with XLH in early childhood, lacked a family history of the disease, and were employed either full- or part-time. A majority did not receive disability payments.

**Table 1:** Demographics.

**Data**	**Frequency**
**Age in years, mean (SD)**	49.1 (13.4)
**Sex assigned at birth**	
**Female**	11/15
**Male**	4/15
**Race, *n* (%)**	
**African American**	3/15
**White**	11/15
**No response**	1/15
**Age at time of diagnosis in years, mean (SD)**	3.4 (4.9)
**Family history of XLH**	
**Yes**	6/15
**No**	8/15
**Unsure**	1/15
**Employment status**	
**Full time**	5/15
**Part time**	5/15
**Retired**	1/15
**Unemployed**	4/15
**Receives disability payments**	
**Yes**	5/14

Abbreviation: XLH, X-linked hypophosphatemia.

Intake survey data related to XLH-related symptoms and therapies are summarized in Table 2. Many participants reported bowing of the legs (100%), bone pain (100%), joint pain (100%), muscle pain (86.7%), and tooth pain (80.0%). Most participants were also diagnosed with degenerative osteoarthritis (80.0%), enthesophytes (60.0%), fractures or pseudofractures (66.7%), spinal stenosis (53.3%), or tooth abscesses (80.0%). The majority of participants occasionally have difficulty walking short distances (40.0%), but always find it difficult to walk long distances (73.3%) and 46.7% use a device to assist with walking. Participants also experience limited ROM in the lower extremities (86.7%), upper extremities (53.3%), and neck or spine (53.3%).

**Table 2:** Self-reported intake survey.

**Data**	**Frequency**
**Burosumab treatment (+/-) and diagnosis**	
**Burosumab (+) Genetic testing/family history**	13/15
**Burosumab (−); Genetic testing/family history**	2/15
**Corrective surgery as a child**	
**Yes**	9/15
**No**	6/15
**Children (offspring) diagnosed with XLH**	
**Yes**	3/15
**No**	12/15
**Short stature**	
**Yes**	13/15
**No**	2/15
**Bowing of the legs**	
**Yes**	15/15
**In-toeing (internal tibial torsion)**	
**Yes**	5/15
**No**	10/15
**Bone pain**	
**As an adult**	15/15
**As a child**	13/15
**Joint pain**	
**As an adult**	15/15
**As a child**	14/15
**Muscle pain**	
**As an adult**	13/15
**As a child**	10/15
**Tooth pain**	
**As an adult**	12/15
**As a child**	5/15
**Diagnosis of comorbid conditions**	
**Arthritis**	12/15
**Enthesophytes**	9/15
**Fracture/pseudofractures**	10/15
**Spinal stenosis**	8/15
**Tooth abscesses**	12/15
**None of the above**	2/15
**Difficulty walking short distances**	
**Always**	3/15
**Occasionally**	6/15
**Rarely**	5/15
**Never**	1/15
**Difficult walking long distances**	
**Always**	11/15
**Occasionally**	4/15
**Rarely**	0
**Never**	0
**Uses device to assist with walking**	
**Yes**	7/15
**No**	8/15
**Has been under the care**	
**Received care by a physical therapist**	15/15
**Received care by an occupational therapist**	4/15
**Received care by a mental health provider**	10/15
**Has limited ROM**	
**Limited neck or spine ROM**	8/15
**Limited upper extremity ROM**	8/15
**Limitedlower extremity ROM**	13/15
**None of the above**	1/15
**Experiences pain when walking**	
**Always**	8/15
**Occasionally**	7/15
**Struggles to walk**	
**Always**	6/15
**Occasionally**	3/15
**Rarely**	2/15
**I can walk but I tire easily**	4/15
**Ability to perform daily tasks**	
**I can perform daily tasks with no problem**	2/15
**I can perform most of my daily tasks with no problem**	4/15
**I can perform some of my daily tasks with no problem**	6/15
**I struggle to perform daily tasks**	3/15
**Uses adaptive equipment to perform daily tasks**	
**Always**	0/15
**Occasionally**	7/15
**Rarely**	3/15
**Never**	5/15
**Pain medication**	
**Over-the-counter**	15/15
**Prescription**	12/15
**How often do you have pain**	
**I always have pain**	8/15
**I have pain frequently**	6/15
**I have pain occasionally**	1/15
**When do you experience pain**	
**Always**	1/15
**At rest**	7/15
**Randomly**	1/15
**With activity**	13/15
**Other: with weather changes**	1/15
**How often do you feel anxious**	
**Most of the time**	2/15
**About half the time**	5/15
**Sometimes**	7/15
**Never**	1/15
**How often do you feel depressed**	
**Most of the time**	1/15
**About half the time**	1/15
**Sometimes**	12/15
**Never**	1/15
**How often do you feel hopeless**	
**Most of the time**	0/15
**About half the time**	2/15
**Sometimes**	10/15
**Never**	3/15
**Childhood treatment**	
**Calcitriol**	2/15
**Phosphate and calcitriol**	11/15
**Calcium and calcitriol**	1/15
**No response**	1/15

Abbreviations: ROM, range of motion; XLH, X-linked hypophosphatemia.

Approximately half (53.3%) of participants noted that they always have pain and all (100%) manage pain using a combination of over-the-counter pain medication and prescription medication. Most participants reported that they are currently taking burosumab (86.7%). Non-pharmacological interventions included surgery (26.7%), and 1 patient reported the use of steroid injections and transcutaneous electrical nerve stimulation unit. All participants had utilized physical therapy as an intermittent treatment modality.

When asked about mood, most individuals expressed that they sometimes feel anxious (46.7%), depressed (80.0%), or hopeless (66.7%).

### SF-36 survey data

**Figure 1:**
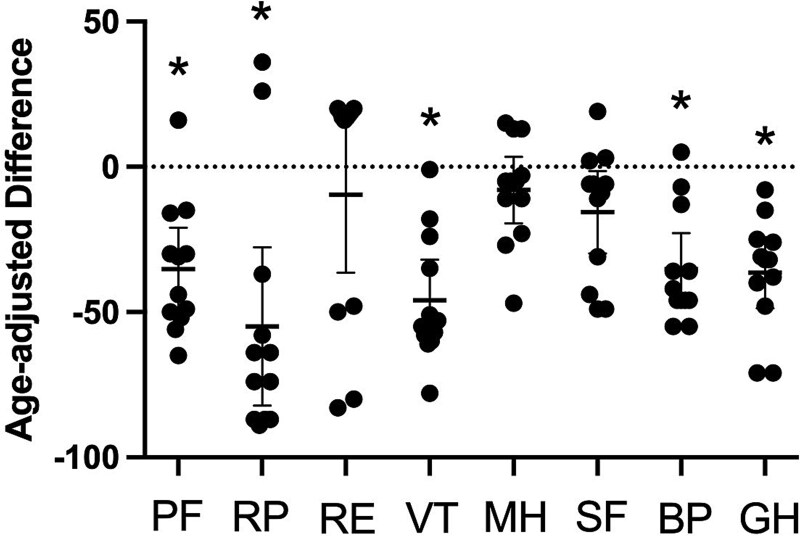
SF-36 age-adjusted differences between the general age-matched population and subjects with XLH. Significant differences are seen in the physical parameters of the health assessment survey (^*^p<.001). Abbreviations: BP, bodily pain; GH, general health; MH, mental health; PF, physical functioning; RE, role limitations due to emotional problems; RP, role limitations due to physical health; SF, social functioning; VT, vitality; XLH, X-linked hypophosphatemia. Clinically meaningful differences for physical disability were calculated using Cohen’s *d*.

Of the 15 subjects, 2 did not complete the SF-36. Age-adjusted differences for SF-36 responses were lower than the general national norm populations^28^ in all domains of health-related quality of life (Figure 1). Significant differences notably related to physical status and pain (*p*<.001; physical functioning [PF], role limitations due to physical health [RP], vitality [VT], bodily pain [BP], and general health [GH]). The XLH group (*n* = 13) demonstrated significantly lower scores compared with the control group, with a large effect size (Cohen’s *d* = 0.80).

### Thematic analysis

All participants completed the interviews aimed at understanding the experience of XLH-related pain and gauging interest and informing the creation of a coping tool to help manage pain. Patients were queried on several broad categories that included the experience and impact of pain and fatigue, pain management strategies, access to services, understanding the experience of talking about pain with healthcare providers, family, friends, and employers, future support as their disorder progresses, advice for others with XLH, and learning and knowledge related to the origins of their pain and ways to manage it (Table 3). Qualitative analysis of the interview transcripts revealed 10 themes related to participants’ current experiences living with XLH-related pain (Table 4).

**Table 3:** Interview guide.

Do you experience pain related to your XLH?
Where is the pain located right now?
Where are other locations that your pain frequently occurs?
What is the source of your pain, if you know? (Some examples include general bone pain, acute bone pain from a fracture, arthritis, or nerve pain)
Does your pain affect your daily activities and/or quality of life?
How does your pain affect these other aspects of your life?
Does any instance of pain interfere more with your daily activities and/or quality of life as compared with others?
If some locations/types of pain cause more of an adverse effect than others, which ones would you particularly like help with?
Which locations/types of pain do you feel you can manage on your own?
On a scale of 1 to 10, 1 being no pain and 10 being the worst pain you have ever experienced, what is the highest level of pain you are able to withstand without interfering with your daily activities and/or quality of life?
Using the same scale as above, what level of pain usually causes you to seek treatment or take medication to manage your pain?
Are you aware of anything you do or experience that worsens your overall pain or any individual locations/forms of pain? If so, what is it?
Do you experience fatigue or tire easily?
How does this change on a daily or weekly basis, if at all?
Is it seemingly random or is it associated with an identifiable cause? If it has an identifiable cause, what is it? (Some examples may include physical activity, insomnia related to XLH (restless legs from burosumab), treatment levels, peak pain levels, depression, and side effects of pain meds)
What do you currently do to manage your fatigue?
What do you currently do to manage your pain?
Have you received physical therapy for XLH-related pain?
Please describe your experience and how long you received treatment
Were there any factors that contributed to either your continuing or stopping physical therapy?
Can you tell me about any difficulties you have had accessing services to help you deal with the impact of chronic pain on your daily life?
What services would you like to have available to help you manage your pain?
What is it like to talk about your pain to your healthcare providers? Family? Friends? Employers?
How do you think your pain will affect you in the future?
What kind of services would be helpful for you to deal with your pain as your disorder progresses?
If you had to tell someone else about how to manage or cope with their XLH-related pain, what would you tell them?
How have you learned about your pain and how to manage it?
How would you feel about getting information on ways to better manage your pain from a self-management mobile app?
What features would you find useful in a self-management pain app?
Is there anything else you would like to tell me related to the pain you experience before we end today that we have not covered?

Abbreviation: XLH, X-linked hypophosphatemia.

**Table 4:** Thematic analysis.

**Theme**	**Frequency**
**1. Chronic and variable pain**	15/15
**2. Impact on mobility and daily life**	15/15
**3. Pain management techniques**	15/15
**4. Healthcare and accessibility challenges**	15/15
**5. Emotional and Psychological Impact**	12/15
**6. Desire for improved information and support**	15/15
**7. Challenges with inactivity and the need to keep moving**	11/15
**8. Navigating Healthcare Services**	11/15
**9. Long-term outlook and concerns**	14/15
**10. Advocacy and awareness**	14/15

All participants (15/15) experience chronic pain that varies significantly in intensity and location, greatly impacting their physical capabilities and quality of life. This persistent pain and its associated symptoms severely restrict mobility and daily activities, leading to changes in lifestyle, employment, and a need for support with routine tasks. To manage their pain, all patients report employing a variety of strategies, including prescription and over-the-counter medications, physical therapy, lifestyle adjustments, and alternative therapies such as yoga and assistive devices. Accessing appropriate medical care poses a challenge for all patients, compounded by limited provider knowledge of XLH and geographic isolation.

The chronic pain and mobility limitations associated with XLH contribute to substantial emotional and psychological impacts, with most patients (12/15) experiencing feelings of isolation, depression, and the strain of managing a poorly understood condition. Social isolation and dependency are also prominent, with most patients reporting increased reliance on others, which affects personal relationships and often leads to frustration and helplessness. Patients (15/15) express a strong desire for improved information about XLH, better treatments, and a supportive community, noting that digital tools, such as mobile apps, could help facilitate this.

Maintaining physical activity within individual limits is crucial yet challenging, as most patients (11/15) find that inactivity worsens pain and immobility. However, the complexity of XLH makes navigating healthcare services challenging, particularly in obtaining essential treatments like burosumab and managing insurance issues. Additionally, the majority of patients (14/15) are concerned about the long-term progression of XLH and its impact on their future health and mobility, highlighting the need for dependable palliative care and comprehensive management.

Finally, most patients (14/15) emphasize the need for advocacy to raise awareness about XLH among the public and healthcare professionals, which could improve care and deepen understanding of the disease. Representative quotes from each thematic area are summarized in Table 5.

**Table 5:** Thematic representative quotes.

**Chronic and variable pain**
*Today, I may not have any pain at all. Those days are few and far between. And tomorrow I may be sitting in my lounge chair the whole day. And then another day I might have half a day’s activity. And another day I might be able to conduct my personal things like cleaning house or washing clothes. But by the end of the day I’m just totally in pain. So it’s totally random.*
**Impact on mobility and daily life**
*It’s very hard to ignore, the pain also comes with stiffness. So then I’m somewhat limited in my range of motion of my hips, my back, my ankles for sure. But it’s more so the pain is hard to focus on anything else. And ultimately, that’s what keeps me from being able to do a lot of the things that I want to do.*
**Pain management techniques**
*Some medicine helps but I relied so heavily on NSAIDs for the last 3 years. My endocrinologist has told me I need to stop taking them so I don’t damage my other organs, but I don’t. You know they help, but it’s like you’re stuck between what you can do. So that’s very frustrating.*
**Healthcare and accessibility challenges**
*As far as finding an orthopedic specialist, I have not found one since I left (my town) 13 years ago. Now I’m told, “We don’t know why you’re in our office because your bones are not broken at the moment, and there is nothing we can do.” I’m sorry I’ve had to deal with that, so I’ve kind of just stopped looking for doctors. The last one wasn’t really receptive to the questions that I had and was very dismissive. So I just kind of dropped it.*
**Emotional and psychological impact**
*The pain doesn’t only affect you physically, it affects you mentally, and you know, sometimes you lose friends, you lose family, you lose a lot of enjoyment in life because of what you can’t do. I can in my head for this month to go to five or six functions and I’ll be lucky if I feel up to going to two. And sometimes people don’t understand that. And I don’t like to complain about it either because sometimes people get used to hearing people’s sob stories to where they almost feel as if everything is an excuse.*
*It’s very important to me to have a pain psychologist to talk to. They are very far and few between. I think that would be so vital for all of us dealing with chronic pain. I miss that actually because I don’t want to overburden my friends and family… I can talk about it to my healthcare providers, but talking about it to my friends and family makes me makes me cry, so I don’t want to overburden them.*
**Desire for improved information and support**
*Reaching out to people who do have XLH, I think that’s important to know that you’re not imagining this weird pain that doctors look at you like you’re crazy. When you mentioned, you know the whole body thing, that it’s everywhere at the same time and there’s no visible source of it, you know, getting validation. That goes a long way, I think, in being able to say, “Oh, yeah, that’s just my stupid XLH acting up” so, even though it hurts, at least I know the pain is real right? There’s a certain comfort in that.*
**Challenges with inactivity and the need to keep moving**
*Basically everything makes the pain somewhere worse at some point. But I also notice that if I don’t do things, if I’m not as active as I can possibly be as often as I possibly can then not only do I have more pain, but I also have more stiffness. I lose that flexibility and strength that I do have. So I’m always trying to weave a balance of what do I think I can manage to do to keep that without making things worse or working myself into a flare up.*
**Navigating healthcare services**
*So before I became an adult, and my mom was my primary caretaker, she experienced various doctors saying that they aren’t familiar with that (XLH), so they would refuse to see me. And then insurance issues were always a problem. They pretty often threatened to not cover even basic medications that I took as a child.*
**Long-term outlook and concerns**
*I don’t know, but I’m really concerned about how pain will affect my future. I’m almost afraid because it’s a progressive disease. And I have seen the progression in myself. I’m concerned that the pain may stop me from doing much of anything. And so I’m not excited about the future as far as pain goes. I already have a power wheelchair and a rollator and a cane and around the house most of the time I walk independently... I have seen the progression in myself and I’m really anxious about it you know.*
*I’m not sure, and that scares me. I think that most of all, that scares me because I’m 57, and I think that I wonder what 10 years is gonna feel like. So there’s a fear of the future for me but I’m hopeful you know. I’ll just take it day to day and and see what happens. But yeah, I can only imagine, it’s gonna get worse, but I don’t know.*
**Advocacy and awareness**
*The emergency department doctor did feel very dismissive, very much so like he thought I was drug seeking or something when I didn’t even ask for controlled substances or anything. And then, as an adult, when I’m trying to find new doctors who say “I don’t know what that is- sorry.” Find somebody else kind of thing.*

Abbreviation: XLH, X-linked hypophosphatemia.

### Digital tools for pain management

In addition to understanding the experience of living with XLH-related pain, another goal of this study was to inform the creation of a pain-coping tool that has been utilized in other chronic and progressive disorders.^29–33^ All participants (15/15) expressed interest in using a pain-coping application. Participants identified several features they would find valuable in a mobile application designed to help manage pain associated with XLH. Key functionalities included tools for tracking pain by specific location and type, as well as managing medications. They also emphasized the importance of tracking physical therapy and exercise routines, maintaining a diary to log pain intensity and its impact, and having access to educational resources tailored to XLH. Additionally, participants expressed interest in community and support features that foster a sense of connection, along with integration capabilities that allow healthcare providers to monitor and respond to patient data. Detailed symptom logging and analysis, as well as resources focused on emotional and psychological support, were also considered essential to support comprehensive pain management.

## Discussion

While several studies have looked at the pain and functional limitations that individuals living with XLH deal with on a daily basis,^3^^,^^6^^,^^7^^,^^12^^,^^17^ this is the first to utilize qualitative analysis to capture their stories and to inform the creation of a pain management tool. Our analysis showed that while individual experiences may differ, there is a commonality within the pain and challenges they face, especially in the areas of chronic and variable pain and its impact on daily life, the variety of pain management strategies used to manage symptoms, healthcare and accessibility challenges, and the resultant emotional and psychological impact. In addition, participants expressed the desire for improved information and support when it comes to their diagnosis, leading to an interest in digital tools for pain management.

When looking at patient reported outcome measures, individuals with XLH often report diminished health-related quality-of-life measures. In a study looking at the lifelong impact of XLH, participants scored greater than 1 SD below the US general population norm in the physical component summary as a whole, along with lower scores in physical functioning, role-physical, bodily pain, and general health subscales.^17^

These findings provide strong evidence that patients with XLH endure a lifelong burden of pain, primarily linked to the impact of phosphate-wasting on musculoskeletal health. The most highly reported pain related to XLH in both children and adults is described as bone pain and joint pain.^17^ The origin of bone pain in XLH is not well studied, although it is known that single nerve fibers are found in Haversian canals and that the periosteum and bone marrow are innervated with both peptidergic and non-peptidergic afferent nociceptive neurons.^34^ Mechanical activation of these afferents could be related to pseudofracture or looser zones, a hallmark of osteomalacia. Current data suggest that fracture healing reported with the use of burosumab improves bone pain for patients with XLH.^22^^,^^35^ For joint pain, the most relevant insights can be drawn from evidence on the origins of pain in osteoarthritis among adults without XLH. OA pain, arising from stimulation of primary afferent nociceptive nerves of the synovium, most often occurs because of mechanical stimuli associated with ADLs. OA pain is also subject to plasticity of the pain response, which involves both peripheral and central sensitization, leading to increased pain sensitivity and persistent pain without mechanical load. In addition, OA pain is influenced by the release of inflammatory molecules that can activate or sensitize nociceptors within the joint.^36^ The tooth pain in XLH is likely due to the activation of nociceptive pathways because of dental caries or periodontal abscesses and associated inflammation or erosion of the exposed dentine.^4^^,^^11^^,^^19^^,^^29^^,^^37^ More poorly studied is the pain related to mineralizing enthesopathies, an aneural tissue that may be subject to nerve remodeling in the adjacent subchondral bone.^36^ The enthesopathy of XLH is dominated by fibrocartilaginous insertion sites that are anatomically associated with joints of the upper and lower body and spine, making it difficult to determine if the mineralization of the enthesis is involved only in restricting ROM or if the pain is mediated by the proximal joint.^5^ Due to the complex and evolving nature of pain in XLH, and lack of effective treatments, a move toward personalized pain management strategies for disorders like XLH is needed.

All individuals interviewed expressed interest in a mobile application specifically designed for the management of XLH-related pain. Most participants commented on the importance of movement and staying active within their individual limits. Of the common themes related to a pain management application, we are particularly interested in incorporating a physical therapy and exercise tracking component to tie into these challenges with inactivity and the need to keep moving. Using these data, we plan to integrate the only XLH-specific physical therapy (PT) program that has been developed.^38^ This evidence-based PT program was designed to address the significant progressive physical disability related to XLH and improve confidence in performing ADLs under the guidance of a physical therapist. When asked, participants were unaware of this program and many expressed interest in incorporating this into their treatment. This includes the incorporation of self-management pain tools like mobile phone applications that can mitigate barriers such as limited healthcare providers and geographical access.^32^

One such intervention is iCanCope with Pain, a mobile application founded in cognitive behavioral therapy that employs a contingency management approach that rewards engagement and compliance with positive reinforcements. This intervention has shown efficacy for managing pain in youth living with sickle cell disease and juvenile idiopathic arthritis and in adults with neurofibromatosis type 1, a rare autosomal dominant genetic disorder that often presents with chronic pain.^29-33^ The app utilizes a self-management platform, allowing users to set SMART goals and track factors like symptoms, mood, and sleep, in addition to providing detailed pain education, social support, and pain self-management tools. As next steps, data from this current study will allow us to better inform the creation of a digital pain management tool for XLH.

### Study limitations

One limitation of this study for portions of the intake and SF36 surveys is the reliance on patient recall, which is subject to recall bias. This study is also limited by sample size, although data saturation of qualitative data was achieved within the first round of patient interviews, speaking to the strikingly similar physical phenotype in affected XLH patients.^5^

## Data Availability

The data underlying this article cannot be shared publicly due to the privacy of individuals that participated in the study. The data will be shared on reasonable request to the corresponding author.
